# Psychological assessment of mothers and their daughters at the time of diagnosis of precocious puberty

**DOI:** 10.1186/s13633-015-0001-7

**Published:** 2015-03-16

**Authors:** Melissa J Schoelwer, Kelly L Donahue, Kristina Bryk, Paula Didrick, Sheri A Berenbaum, Erica A Eugster

**Affiliations:** Pediatrics, Section of Pediatric Endocrinology, Riley Hospital for Children, 705 Riley Hospital Drive, Room 5960, Indianapolis, IN 46202 USA; Pediatrics, Section of Adolescent Medicine, Indiana University School of Medicine, Indianapolis, IN USA; Psychology and Pediatrics, The Pennsylvania State University, University Park, PA USA; Psychology, The Pennsylvania State University, University Park, PA USA

**Keywords:** Central precocious puberty, Premature adrenarche, Early normal puberty, Psychopathology

## Abstract

**Background:**

Concerns about psychological distress are often used to justify treatment of girls with precocious puberty, but there is little evidence to support these concerns. The extent to which psychological problems are associated with central precocious puberty (CPP) compared with other forms of early puberty in girls has likewise not been established.

**Methods:**

Girls presenting with untreated CPP, premature adrenarche (PA) or early normal puberty (ENP) were recruited from our pediatric endocrine clinic along with their mothers. Child psychological adjustment was assessed by child self-report and parent report. Parent self-reported personality, anxiety, and depression were also assessed. Differences between groups were explored using one-way ANOVA and Dunnett’s T3 test.

**Results:**

Sixty-two subjects (aged 7.5 ± 1.4 years, range 4.8-10.5) were enrolled, of whom 19 had CPP, 22 had PA, and 21 had ENP. Girls with ENP were significantly older (8.9 ± .9 years) than girls with CPP (6.9 ± 1.1 years, p < .001) and PA (6.6 ± 1.0 years, p < .001). Girls with PA had significantly higher BMI z-scores (1.7 ± .8) than girls with CPP (1.1 ± .6, p = .01) and ENP (1.2 ± .6, p = .04). More girls with PA and ENP were from racial minorities (47% and 50% respectively) than girls with CPP (32%). No group differences were found for any child measure of psychological adjustment. However, mothers of girls with PA scored significantly higher than mothers of girls with ENP on one measure of depression (p = .04) and stress (p = .01).

**Conclusions:**

While mothers of girls with PA report increased psychological distress on some measures, no differences in psychological adjustment were found at baseline amongst the girls themselves. Whether these results will change as puberty progresses in the PA and ENP groups or with treatment of CPP is unknown. Long-term prospective studies are needed in order to further investigate psychological correlates of early puberty in girls.

## Background

Central precocious puberty (CPP) is due to early activation of the hypothalamic-pituitary-gonadal (HPG) axis prior to the age of 8 in girls or the age of 9 in boys and results in the presence of secondary sexual characteristics and accelerated somatic growth. CPP is much more common in girls compared to boys and is typically idiopathic in nature [1]. Medical intervention is warranted in children with CPP to prevent short adult height secondary to early epiphyseal closure. Treatment of CPP with gonadotropin-releasing hormone analogs (GnRHa) has been found to be safe and effective [2,3]. However, a predictable increase in adult height occurs primarily in girls under the age of 6 years [4,5,3]. Despite this, girls under the age of 6 comprise only a small number of those who are treated.

Another justification for treatment of girls with CPP involves alleviation of psychological distress [6,7]. This concept is primarily extrapolated from studies showing higher rates of problem behaviors and lower academic achievement during adolescence in girls experiencing menarche that is early but still within the normal range [8-12]. The few studies of psychological function in girls with CPP have yielded inconsistent results and have been limited by methodological problems including small sample sizes, retrospective designs, insufficient psychological assessment, and lack of controls [13,14,7]. Although parents of daughters with early puberty may have strong reactions to their child’s physical development and are worried about early sexual behavior and peer rejection, it is essential to separate a parent’s distress from a girl’s own distress [7,6]. To what extent girls with CPP experience psychological problems and whether treatment alleviates these issues remains unclear.

Premature adrenarche (PA) is another form of early puberty that is considered a common variation of normal development. However, there are some reports of higher rates of psychopathology in girls with PA compared with their counterparts with on-time adrenarche [15,16]. Unlike girls with CPP who are exposed to elevated estrogens, girls with PA are exposed to mildly elevated androgens earlier than their peers. How girls with PA compare to girls with CPP with regards to psychological profiles is unknown.

In this study we examined the extent to which psychological distress is present before treatment in girls with CPP compared to girls with PA and girls with early normal puberty (ENP). We likewise examined the psychological characteristics of the mothers of the girls. We hypothesized that girls with CPP would demonstrate a distinct psychological profile compared to girls with PA and ENP due to the effects of early hormonal exposure on the brain and social experiences caused by a mismatch between chronological age and physical development.

## Methods

Girls who were newly diagnosed with CPP, PA, and ENP were prospectively recruited from the pediatric endocrine clinic at Riley Hospital for Children along with their mothers. The study was approved by the Indiana University School of Medicine Institutional Review Board. Written informed consent was obtained from all mothers and assent was obtained from all girls aged 7 and older. All patients were evaluated and diagnosed by a pediatric endocrinologist. Inclusion criteria for CPP consisted of pubertal onset prior to 8 years of age along with biochemical confirmation of HPG axis activation (random ultrasensitive LH > 0.3 mIU/ml or GnRHa stimulation test with peak LH > 4 mIU/ml). Inclusion criteria for PA included age between 5 and 8 years along with clinical, radiographic and biochemical features consistent with this diagnosis. Inclusion criteria for ENP included age between 7.5 and 10 years as well as clinical and radiographic evidence of central puberty. Exclusion criteria for all groups included inability to speak English, prior treatment with a GnRHa, and developmental delay or significant behavior problems precluding cooperation with an interview. Psychological assessments of the girls and their mothers were performed on the same day or within a few weeks of the initial clinic visit.

### Child psychological functioning (self-report)

Girls completed the Children’s Depression Inventory (CDI) a 26-item scale measuring depressive symptoms experienced during the previous two weeks [17]. Responses were measured on a three-point scale. All items were coded such that a higher score indicated a higher level of depressive symptoms, with the sum of items used in analyses. The CDI was completed only by participants ages 7 and above.

Girls also completed the Harter Pictorial Scale of Perceived Competence and Social Acceptance for Young Children, which assesses a child’s perceived abilities in the domains of cognitive and physical competence and peer and maternal acceptance [18]. The Harter Scale consists of 24 items measured on a 4-point scale. All items were coded such that a higher score indicated a high level of perceived competence or acceptance, with the mean of items from each scale used in analyses. Participants completed either the preschool/kindergarten or 1st/2nd grade version of the scale.

### Child psychological functioning (parent-report)

Mothers completed either the Child Behavior Checklist (CBCL) for ages 1 ½ to 5 years or the CBCL for ages 6 to 18 years [19,20]. The CBCL measures children’s behavioral and emotional problems and yields internalizing and externalizing subscales as well as a total problems score. The CBCL for ages 1 ½-5 years consists of 99 items assessing the child’s behavior within the past two months using a 3-point scale, while the CBCL for ages 6–18 years consists of 113 items assessing behavior within the past six months using the same 3-point scale. Population-normed T-scores were used for each participant.

### Parent psychological functioning (parent-report)

Mothers completed the NEO Five-Factor Inventory-3 (NEO-FFI-3), a 60-item questionnaire measuring five domains of personality: neuroticism, extraversion, openness, agreeableness, and conscientiousness. Responses were measured on a 5-point Likert scale. All items were coded such that a higher score indicated a higher degree of the relevant personality trait. The sum of items from each of the five scales was used in analyses.

Mothers also completed the Depression, Anxiety, and Stress Scale (DASS), a 42-item measure of these three negative emotional states experienced during the past week [21]. Responses were measured on a 4-point scale, with items corresponding to depression, anxiety, and stress subscales. All items were coded such that a higher score indicated a higher level of the emotional state, with the sum of items from each scale used in analyses.

Finally, mothers completed the Positive and Negative Affect Schedule (PANAS), a 20-item measure of emotions experienced within the past week [22]. Responses were measured on a 5-point scale, with items corresponding to positive affect and negative affect scales. All items were coded such that a higher score indicated a higher level of positive or negative affect.

### Statistical analysis

One-way analyses of variance (ANOVAs) tests with planned contrasts were conducted to examine differences between the CPP, PA, and ENP groups with regard to age and BMI *z*-score. Chi-square tests were used to test differences between groups with regard to racial minority status. Group differences in child and parent psychological functioning were examined using one-way ANOVA and Dunnett’s T3 test for pairwise comparisons. For all tests, Type I error was set at .05. Analyses were performed using SPSS 22.

## Results

Sixty-two subjects were enrolled, of whom 19 had CPP, 22 had PA, and 21 had ENP. Patient characteristics are outlined in Table 1. The girls had an average age of 7.5 ± 1.4 years (range 4.8-10.5). As expected, girls with ENP were significantly older than girls in the other groups (p < .001) and girls with PA had significantly higher BMI z-scores than girls with CPP and ENP (p = .02). More girls with PA and ENP were from racial minorities than girls with CPP (p = .03). Two girls in the CPP group were post-menarchal whereas 7 girls in the ENP group had reached menarche.

**Table 1:** **Patient characteristics**

	**CPP n = 19**	**PA n = 22**	**ENP n = 21**
Age^a^ (mean years ± SD)	6.94 ± 1.1	6.60 ± 1.0	8.80 ± 0.9
Race^b^ (% non-Caucasian)	32%	46.67%	50%
BMI^c^ (mean z-score ± SD)	1.09 ± 0.6	1.66 ± 0.8	1.22 ± 0.6
Post-menarchal (percentage)	2 (11%)	0	7 (33%)

^a^Results of planned contrast indicated significant difference between ENP group and CPP and PA groups, p < .001, equal variances assumed.

^b^Results of chi-square test indicated group differences in percentage of racial minorities, p = .03.

^c^Results of planned contrast indicated significant difference between PA group and CPP group, p = .01, as well as PA group and ENP group, p = .04, equal variances not assumed.

Results on tests of psychological adjustment are displayed in Table 2. Child self-report measures were not significantly different among the three groups. No relationship was seen between BMI z-scores and test results in girls with PA. It is worth nothing that mean scores for all groups on these measures were within the published normative ranges. However, mothers of girls with PA scored significantly higher than mothers of girls with ENP on one measure of depression (p = .04) and stress (p = .01).

**Table 2:** **Psychological assessments**

	**CPP**	**PA**	**ENP**	**p value**
**Child assessments**				
CDI	n = 9	n = 4	n = 13	0.14
(in subjects ≥ age 7)	7.78 ± 6.8	13.0 ± 11.5	5.31 ± 4.3	
Harter scale	n = 18	n = 22	n = 19	
Cognitive competence	3.68 ± 0.3	3.46 ± 0.5	3.49 ± 0.5	0.25
Peer acceptance	3.13 ± 0.7	3.25 ± 0.7	3.17 ± 0.5	0.84
Physical competence	3.31 ± 0.6	3.39 ± 0.6	3.21 ± 0.5	0.57
Maternal acceptance	2.59 ± 0.7	2.83 ± 0.7	2.66 ± 0.6	0.52
CBCL	n = 18	n = 21	n = 19	
Internalizing scale (T-score)	51.72 ± 11.7	49.62 ± 9.9	50.70 ± 9.3	0.82
Externalizing scale (T-score)	51.78 ± 9.8	48.52 ± 11.9	46.89 ± 6.9	0.32
Total problems (T-score)	51.18 ± 10.6	47.75 ± 9.7	48.95 ± 8.6	0.56
**Mother assessments**				
NEO-FFI-3	n = 19	n = 22	n = 19	
Neuroticism	17.26 ± 7.5	19.32 ± 7.2	18.63 ± 5.8	0.63
Extraversion	29.37 ± 5.1	30.27 ± 5.8	30.26 ± 4.6	0.82
Openness	25.79 ± 5.4	24.41 ± 5.5	25.89 ± 4.6	0.53
Agreeableness	32.16 ± 3.8	32.27 ± 3.6	31.95 ± 4.1	0.96
Conscientiousness	34.05 ± 6.2	31.95 ± 8.4	36.00 ± 6.4	0.20
DASS	n = 19	n = 22	n = 20	
Depression	3.89 ± 6.4	4.73 ± 5.8	1.25 ± 1.3	0.04*
Anxiety	3.68 ± 5.9	3.59 ± 3.7	1.55 ± 1.9	0.19
Stress	8.68 ± 7.5	10.86 ± 7.0	4.90 ± 4.2	0.01*
PANAS	n = 19	n = 22	n = 20	
Positive affect	34.37 ± 10.0	33.91 ± 9.9	34.10 ± 6.6	0.99
Negative affect	15.32 ± 6.0	15.91 ± 4.1	14.90 ± 5.7	0.82

*significant difference, PA vs. ENP.

## Discussion

Although frequently touted as a rationale for initiating treatment with a GnRHa, the psychological consequences of precocious puberty in girls are unclear. Results from studies performed to date have been decidedly mixed. While some have found evidence of behavior problems and psychological distress, others have reported positive self-esteem and normal psychosexual function in early-maturing girls [12,23]. Whether treatment with a GnRHa alters the psychological trajectory in girls with early puberty is similarly unclear. Thus, no consensus regarding psychological outcomes in either treated or untreated precocious puberty has emerged thus far [3].

Many studies of girls with early puberty have relied primarily on the CBCL completed by parents or teachers. A strength of our study is that the girls themselves were directly tested rather than solely relying on parental report. Our study is also unique in that we included the two comparison groups of PA and ENP. To our knowledge, no previous study has compared the psychological characteristics of mothers and their daughters with CPP, PA and ENP.

There are speculations that girls with early puberty are likely to suffer from teasing, embarrassment, and social isolation due to looking different from their peers [7]. Although the girls with CPP in our study were physically different from the vast majority of their peers, it is reassuring that they reported normal physical competence and social acceptance. Even within the normal range of puberty there is much interindividual variation in pubertal timing, resulting in same-aged girls significantly differing physically [3]. It is also reassuring that normal psychological scores were found in our girls with ENP. Since more of this cohort had already achieved menarche, they might have been expected to exhibit greater psychological distress than the other groups. In contrast to other studies, no evidence of psychological problems were found in our girls with PA. However, mothers of girls with PA in our study reported increased levels of depression and stress. Whether the psychological differences seen in the mothers of girls with PA are specific to their daughters’ early puberty or due to other factors is unknown.

One possible explanation regarding the mothers of girls with PA is that their psychological distress is secondary to their daughter’s physical changes. Parents of girls with early puberty have been suggested to be worried and embarrassed, and have fantasies of their child dying, having early sexual behavior, and experiencing peer rejection [6]. However, why PA would be more upsetting for mothers than the physical changes that occur in central puberty is unclear. It is also possible that these mothers are experiencing stress secondary to other factors such as family conflict or financial strain. Unfortunately, we did not collect information on socioeconomic status or family composition, so this remains purely speculative.

There are a number of limitations that necessitate caution in interpreting our results. First, although our sample is comparable in size to other studies evaluating psychological characteristics of girls with CPP [13,14], the statistical power is still too low to see anything other than large effects. It is, therefore, still possible that CPP is associated with psychological problems. Second, while we compared girls with CPP to those with PA and ENP, we lacked a control of girls of similar ages who were prepubertal. However, the psychological measures used are based on population norms so it is unlikely that a prepubertal control would have yielded different results. Third, selection bias might have contributed to our results. For example, mothers of girls with PA might be more likely to bring their daughters in for evaluation if they are themselves distressed, although the same could be true for mothers of girls with CPP and ENP. Finally, our psychological assessments were conducted after the girls were evaluated by a pediatric endocrinologist. This might have influenced the mothers’ scores in varying ways, depending on the diagnosis and if they were given reassurance or treatment was discussed. It is worth noting that distress in mothers of girls with PA was not observed on all measures. It is unclear if this reflects low statistical power, Type I error with multiple comparisons, or measurement insensitivity. All girls with CPP are now being treated with a GnRHa while pubertal development in girls with ENP and PA is being allowed to progress. It will be interesting to see if new differences between groups in either the mothers or the daughters emerge over time.

## Conclusions

While mothers of girls with PA report increased psychological distress, no differences in psychological adjustment at baseline were found amongst the girls themselves. This study suggests that concerns about psychological distress are not a definite indication for GnRHa therapy in girls with CPP, at least at the time of diagnosis. Whether or not these results will change over time and with treatment (of CPP) is unknown. Long-term prospective studies are needed in order to further investigate psychological correlates of early puberty in girls.

## Abbreviations

CPP: Central precocious puberty
PA: Premature adrenarche
ENP: Early normal puberty
HPG: Hypothalamic-pituitary-gonadal
GnRHa: Gonadotropin releasing hormone analog
CDI: Children’s Depression Inventory
CBCL: Child Behavior Checklist
NEO-FFI-3: NEO Five-Factor Inventory-3
DASS: Depression, Anxiety, and Stress Scale
PANAS: Positive and Negative Affect Schedule
